# Forty Years in the Making: Reporting on the Co‐Creation of a National Roadmap for Birthing on Country Services for the Best Start to Life

**DOI:** 10.5694/mja2.70282

**Published:** 2026-09-01

**Authors:** Res McCalman, Anneka J. Bowman, Roianne West, Kristie Watego, Jyai Allen, Claire Clack, Sue Kildea, Sue Kruske, Melanie Briggs, Cleone Wellington, Rebecca Coddington, Yu Gao, Isabella Garti, Sascha Kowalenko, Sarah Ireland, Catherine Austin, John D. Boffa, Marah Prior, Kelsie Kahl, Bettina Chaseling, Sarah Khaw, Emily Armstrong, Donna Hartz, Yvette Roe

**Affiliations:** ^1^ Molly Wardaguga Institute for First Nations Birth Rights Charles Darwin University Darwin Northern Territory Australia; ^2^ School of Nursing and Midwifery James Cook University Townsville Queensland Australia; ^3^ Institute for Urban Indigenous Health Windsor Queensland Australia; ^4^ School of Nursing and Midwifery Edith Cowan University Perth Western Australia Australia; ^5^ Department of Health, Disability and Ageing Australian Government Canberra Australian Capital Territory Australia; ^6^ Waminda South Coast Women's Health and Wellbeing Aboriginal Corporation Nowra New South Wales Australia; ^7^ Public Health Division Central Australian Aboriginal Congress Alice Springs Northern Territory Australia; ^8^ Health Programs Division Central Australian Aboriginal Congress Alice Springs Northern Territory Australia; ^9^ Northern Institute Charles Darwin University Darwin Northern Territory Australia; ^10^ School of Nursing and Midwifery University of Western Sydney Penrith New South Wales Australia

**Keywords:** Indigenous health, maternal health, reproductive rights

## Abstract

For over 40 years, First Nations communities in Australia have called for community‐controlled birthing and maternal–infant health programs that restore First Nations‐led, community‐controlled birthing systems grounded in cultural authority and sovereignty. ‘Birthing on Country’ services have demonstrated improved First Nations maternal–infant health outcomes, more babies kept out of the child protection system, and reduced health system costs. Restoration and expansion of Birthing on Country services across regions has been inconsistent, and has lacked support for national implementation. In 2022, a large national gathering (‘Best Start to Life’) was co‐hosted in Mparntwe (Alice Springs). The focus was Birthing on Country service implementation to address inequities in birthing service provision for First Nations communities. A deliverable of this gathering was the *National Roadmap for Birthing on Country Services 2026–2036* (the Roadmap). The co‐creation of the Roadmap maintained enhanced engagement from a range of stakeholders, to produce inclusive and democratic solutions to perpetual perinatal inequities in health. A two‐phased process facilitated national co‐creation where national priorities in the reclamation of First Nations Birth Rights were upheld. The Roadmap builds on an evidence‐informed and First Nations knowledge‐grounded framework, the RISE Implementation Framework. The formation of the Roadmap contrasts against traditional policy creation, transitioning from an evidence‐based proposal to a First Nations‐endorsed national framework, ready for implementation and government adoption. This novel, national strategic approach to upscale Birthing on Country services has received formal endorsement nationally. The Roadmap provides detailed strategies to address structural reform, and its foundational co‐creation process demonstrates that to change practice, researchers must do more than publish evidence in journals for policy change and knowledge translation. The 10‐year key deliverables in the Roadmap are a call to action to recognise Birthing on Country as an inherent sovereign right grounded in First Nations Law and governance.

## 1 Acknowledgement of Country

We acknowledge the Traditional Custodians of the lands, waters and skies across the many Nations on which this work was conceived, developed and will be enacted. We pay our deepest respects to Elders past and present, and to the matriarchs, grandmothers and First Nations women whose cultural authority, knowledge systems and leadership have sustained birthing practices for thousands of generations. We recognise that sovereignty was never ceded, and that First Nations peoples continue to hold enduring relationships with Country that underpin health, wellbeing and the continuation of cultural birthing Law. We honour Arrernte Country in Mparntwe (Alice Springs), where the landmark Birthing on Country Gathering, followed by the Best Start to Life Gathering, took place, and acknowledge the leadership of Aboriginal Community Controlled Health Organisations and First Nations communities who continue to advocate for the restoration of Birthing on Country. This work is grounded in First Nations sovereignty and is guided by the authority of women, Elders, knowledge holders and communities whose governance, decisions and leadership shape the present and future of Birthing on Country.

## 2 Forty Years in the Making

**FIGURE 1:**
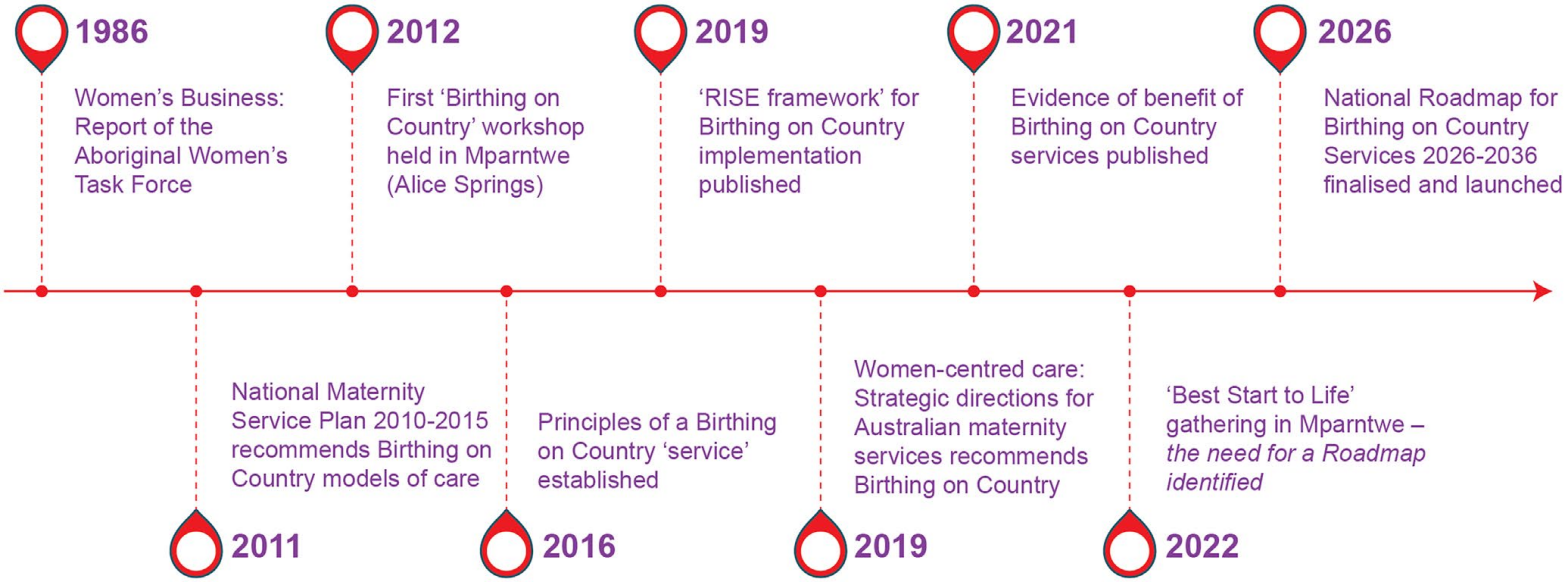
Forty years of advocacy and sustained calls for reclamation of birth rights for First Nations families [3, 4, 5, 6, 7].

This year marks 40 years since the release of the *Women's Business: Report of the Aboriginal Women's Task Force* [1], which was the first time that the Australian Government sought the perspectives of Aboriginal and Torres Strait Islander (First Nations) women in relation to issues that affected them. Women from across the continent articulated their struggles with the growing dominance and expansion of Western‐style, biomedical health and maternity systems [1]. The report called for the reclamation of culturally appropriate birthing and maternal–infant health services, and for them to be based in communities [1]. This call has been sustained by First Nations women across subsequent generations [2, 3, 4, 5, 6] (Figure 1).

Cultural pregnancy and birthing knowledges held by First Nations women have for thousands of generations supported thriving families, communities and cultures [7]. This has been interrupted with 238 years of imposed foreign settler laws, enforced and operating to erase and assimilate First Nations women's cultural identities [8, 9]. First Nations women continue to revive and sustain cultural birthing practices, but this is seldom recognised within mainstream maternity systems, which results in harm and embeds racism within perinatal care [3, 10]. Health inequities persist, such as higher rates of maternal death, perinatal mortality, preterm birth, low birth weight, more sick babies staying in neonatal units [11] and higher reported experiences of birth trauma [12]. Since 2007, Australian Governments have repeatedly committed to ‘close the gap’ in health and wellbeing outcomes, but major barriers to the early success of the subsequent ‘Closing the Gap’ strategies were the absence of First Nations voices, the denial of First Nations governance over health policy design and implementation, and applying medical solutions to a social and political problem [13, 14]. After minimal evidence of progress, the most recent report states that Target 2 (91% of First Nations babies with a healthy birth weight by 2031) is not on track to be met [15], and that both Targets 4 (55% of children are thriving in the early years) and 12 (reduce the rate of overrepresentation of Aboriginal and Torres Strait Islander children [0–17 years old] in out‐of‐home care by 45%) are ‘worsening, and not on track’.

First Nations women want maternity programs that are designed by First Nations people to be clinically and culturally safe, with teams of known care providers [16]. Governments have acknowledged the need to improve maternal–infant health services for First Nations communities in Australia [17, 18, 19], and strategic health policy documents now recommend the implementation of Birthing on Country services [15, 20, 21]. Birthing on Country models of care are holistic, culturally grounded, community‐based and governed maternity services for First Nations women and their families [22]. These models are grounded in relational connections to Country, where land, culture and community are integral to health and wellbeing. Recent evidence shows that Birthing on Country services provide significantly better perinatal outcomes for women and newborns, such as a 38% reduction in preterm birth, improved family engagement with a 54% increased chance of women attending more than five antenatal visits, increased breastfeeding rates [22], preservation of families by reducing newborn removal at birth [23] and health system cost savings of $4810 per mother–baby pair [24]. As a movement to return maternity services to First Nations communities and community control [25], Birthing on Country is a sovereign right for First Nations families [26] and the very essence of what Aboriginal matriarchs have called for, from the 1980s onwards.

In response to academic commentary highlighting persistent postponement in wide‐spread implementation [27, 28], the Federal Government has begun responding to longstanding First Nations advocacy through investment in 10 Birthing on Country services across Australia [15], and has recently committed $44.4 million over the next 4 years to grow these services. Yet without restitution of First Nations community self‐determination of economic, social and cultural strategy, this investment risks stalling and further delaying advocacy for First Nations human rights [29]. A strategic direction informed by First Nations governance, designed to address structural, political and legislative barriers, will ensure government investment into Birthing on Country is channelled appropriately to achieve genuine self‐determination and health equity for First Nations communities. The Molly Wardaguga Institute for First Nations Birth Rights (the Molly Institute) and national supporters have developed a novel evidence‐based strategy to accelerate national scale‐up of Birthing on Country services for First Nations families: the National Roadmap for Birthing on Country 2026–2036 (the Roadmap) [30]. This article describes the national momentum and action in development of this first‐of‐its‐kind Roadmap. As a national, phased approach, the Roadmap is underpinned by the RISE Implementation Framework [25] and involved a large national gathering [6] with ongoing engagement with Aboriginal Community Controlled Health Organisations (ACCHOs), government, non‐government, academic, clinical, policy and community stakeholders.

## 3 The RISE Implementation Framework

**FIGURE 2:**
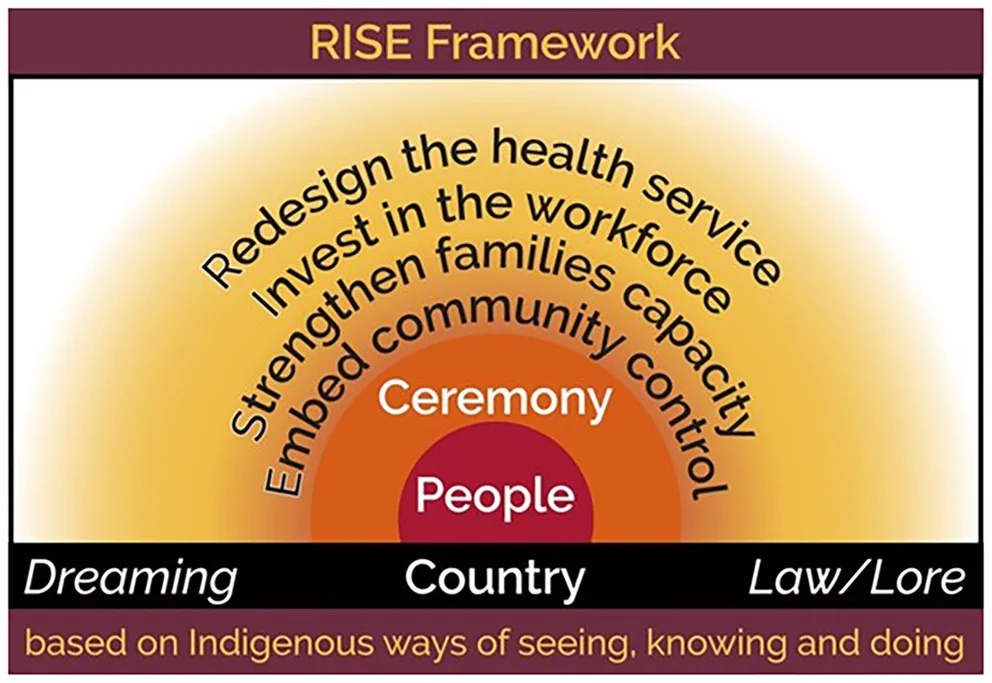
The RISE Implementation Framework [25].

Development of the Roadmap has been underpinned by an evidence‐based Birthing on Country implementation framework, known as the RISE Implementation Framework (Figure 2) [25]. This framework was developed using a synthesis of theoretical, policy and research evidence regarding Birthing on Country services [25]. The four pillars of the RISE Implementation Framework are: (1) Redesign maternity services; (2) Invest in workforce; (3) Strengthen family capacity; and (4) Embed community control. The RISE Implementation Framework provides a phased transition from ‘routine care’ to Birthing on Country best practice, which includes integrated community‐based hubs, choice of birthplace, a culturally and clinically exceptional workforce, strong and resilient families, and First Nations ownership of care provision [25].

## 4 Indigenist Co‐Creation of the Roadmap

As a mechanism for translating evidence into policy and action [31, 32], ‘co‐creation’ fosters enhanced engagement from a broad range of stakeholders and collaborators to produce inclusive and democratic solutions to complex problems [32], which in this instance, are the perpetual perinatal inequities produced by the current maternity system, and slow national implementation of Birthing on Country services [6]. To our knowledge, no other Australian maternal–infant health strategic policy document has been developed using an Indigenist co‐creation methodology [33, 34]. Co‐creation positions First Nations epistemologies, which are usually precluded from health policy planning, as the primary knowledge system, allowing for enhanced innovation and resisting tokenism [35]. Its purpose is not to adjust or adapt systems, but to transform them. Co‐creation, as applied in this Roadmap, is an Indigenous self‐determination methodology as it centres Indigenous authority over the knowledge, decision‐making and governance by making space for First Nations women's leadership [36].

## 5 Co‐Creation Phase 1: Inclusive, Democratic, Active Participation (2022–2024)

**FIGURE 3:**
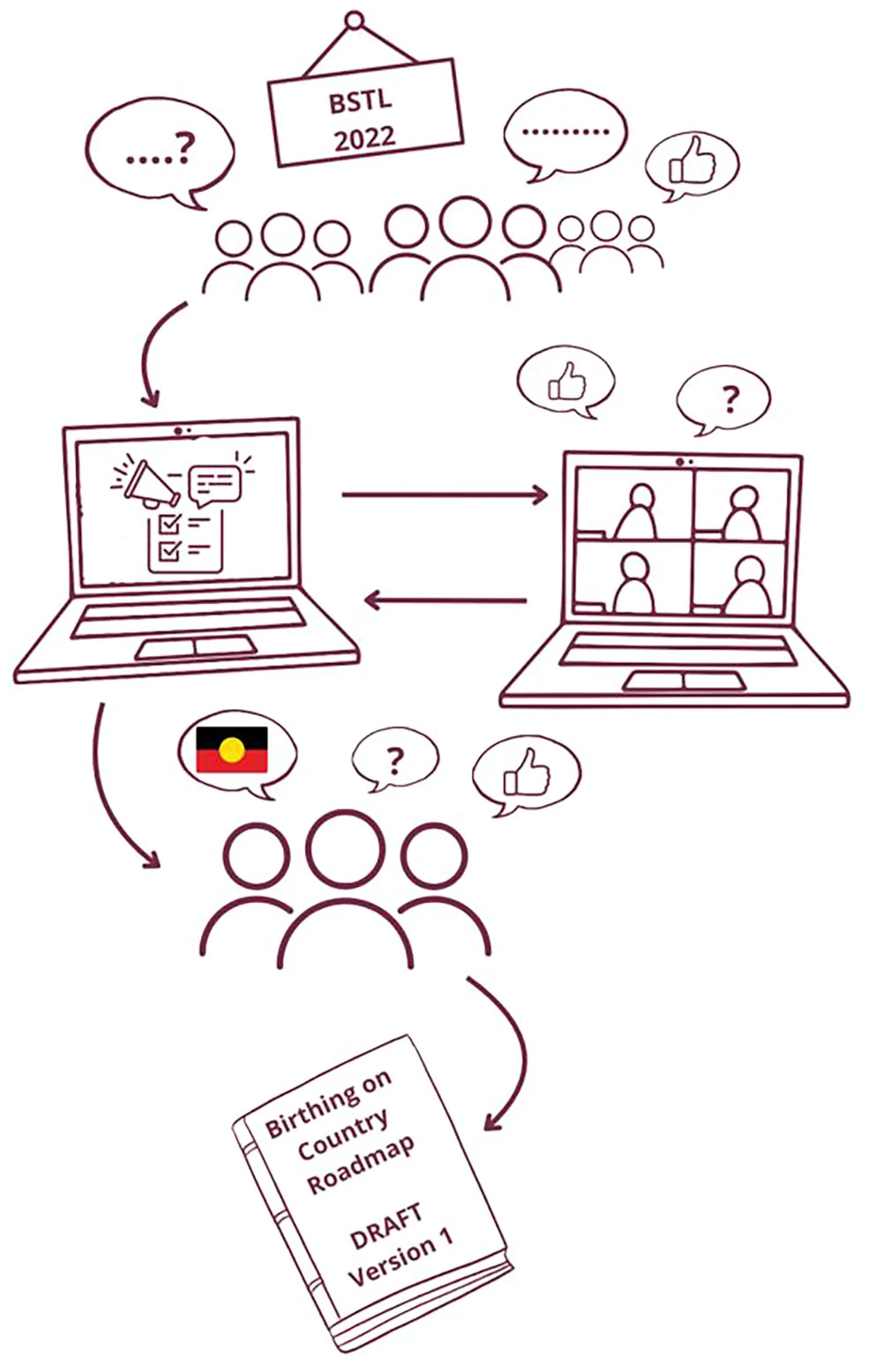
Co‐creation Phase 1: A large national gathering, delegate follow‐up, online workshops, document review, First Nations workshop (Best Start to Life Gathering, BSTL), producing a ‘first draft’.

**FIGURE 4:**
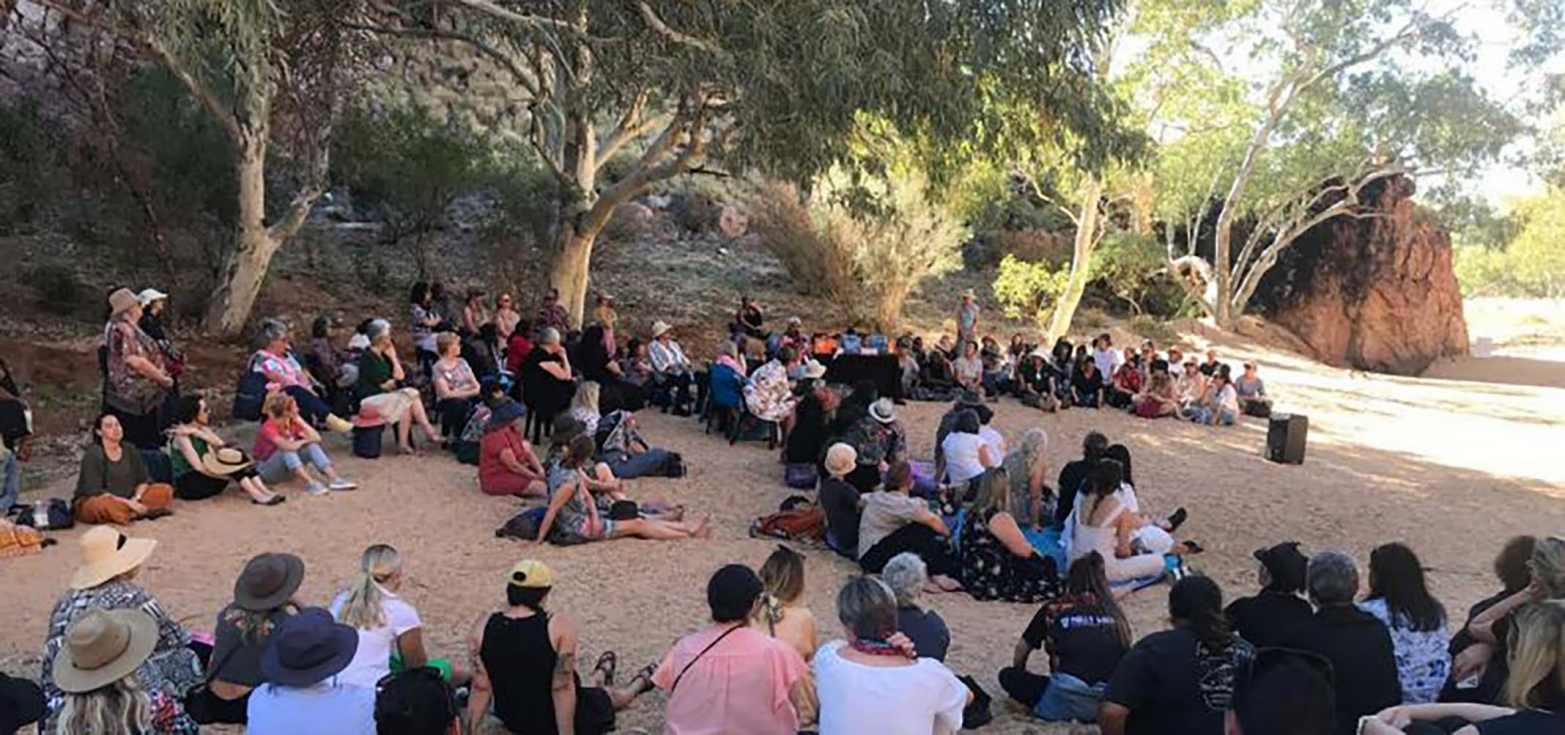
The Best Start to life National Gathering, 2022 [6].

Co‐creation Phase 1 (Figure 3) involved feedback from delegates who attended a large national gathering (The Best Start to Life Gathering) (Figure 4) [6], which was co‐hosted by the Central Australian Aboriginal Congress (CAAC) (an ACCHO in Mparntwe) and the Molly Institute Charles Darwin University. Taking place on Arrernte Country, Mparntwe (Alice Springs), the gathering marked 10 years since an inaugural Birthing on Country national gathering held at the same location [5]. As an organisation that has been at the forefront of the Birthing on Country movement since the 1980s, CAAC has seen decades of community advocacy for the control of birthing and maternal–infant services to be returned to Aboriginal communities [2].

The gathering brought together 245 delegates from 29 organisations, including representatives from ACCHOS, community representatives, family support and Aboriginal health workers, nurses, midwives, doctors, allied health professionals, researchers, educators, service managers, policy advisors, politicians and First Nations leaders. The goal was to identify community priorities, and to understand stakeholder, healthcare provider and research perspectives regarding progress towards improved, effective maternity care for First Nations families and communities [6]. The Best Start to Life Gathering program was strategically designed to align with the four pillars of the RISE Implementation Framework, and the voices of First Nations women's authority and leadership guided all discussions and decisions [37]. After the gathering, each delegate received an invitation via e‐mail to remain engaged through a detailed co‐creation process.

During the weeks following the gathering, key messages from rapporteur summaries, written comments, and 50‐min panel discussions were synthesised to form the basis of a draft Roadmap for Birthing on Country services that included the major activities needed to achieve the desired outcomes over the next 10 years (Table 1). Between January and October 2023, a series of online workshops were scheduled, and stakeholders who had accepted the invitation to remain engaged were able to attend and provide feedback about the contents of this first draft of the Roadmap. Stakeholders across nearly all states and territories of Australia (excluding Tasmania) and from diverse sectors (e.g., numerous health disciplines, ACCHOs, government, community families, women, Elders and cultural groups) provided insight and further refined the Roadmap.

**TABLE 1:** Preliminary themes identified using the RISE Implementation Framework during the Best Start to Life Gathering.

**R**edesign maternity services Women having a First Nations baby have access to local Birthing on Country services Birthing on Country services offer relationship‐based care for the first 1000 days **I**nvest in the workforce ‘Aboriginal midwives for Aboriginal women’ Reimagined First Nations maternity workforce Non‐Indigenous maternity workforce is culturally safe and held accountable for racism **S**trengthen families at the service and structural level Birthing on Country services include holistic programs for whole family Maternity services use trauma‐informed approaches that privilege First Nations knowledge systems and cultural practices **E**mbed First Nations community control Governments address poverty as a social determinant of health Child protection and police come to the table with strengths‐based, trauma‐informed responses Governments resolve barriers to ACCHOs delivering Birthing on Country services Mainstream maternity care embeds mechanisms for clinical and cultural governance of services for First Nations families Translation Birthing on Country Knowledge Hub, Network, Roadmap and Toolkit

Follow‐up working groups focused on single components of the RISE Implementation Framework; that is, ‘Redesign health services’, ‘Invest in the workforce’, ‘Strengthen family capacity’ or ‘Embed community control’. A consortium of First Nations stakeholders provided direction and ownership over the Roadmap draft as strategy was developed. The overall objectives of the Roadmap [30] (p. 7) were positioned at the forefront of development to uphold the priorities from the national gathering. The resultant strategy reflects party consensus and includes informed measurable goals and achievable activities within outlined timeframes (Table 2). Actions aim to transform systems so that First Nations families have access to culturally safe, high quality, relationship‐based and comprehensive primary maternal–infant healthcare [38].

**TABLE 2:** Consensus of desired benefits of the 10‐year Roadmap for Birthing on Country services, across the RISE pillars.

**R**edesign maternal–infant health services for First Nations families	First Nations mothers have an optimal birth experience and are healthy across their lifespan First Nations babies are born strong and are healthy across their lifespan Maternity services receive infrastructure to support demand Birthing on Country improves perinatal outcomes resulting in cost savings for the healthcare system
**I**nvest in clinically and culturally exceptional workforce	First Nations midwives feel valued, safe and well supported, and desire to stay in clinical practice Birthing on Country services have access to First Nations midwives endorsed to provide Medicare‐billable services and work to full scope of practice in any setting Social, cultural and emotional wellbeing of First Nations families is supported during pregnancy, birth and postpartum periods Women having a First Nations baby can access maternity care that is clinically safe, feels culturally safe and is free from all forms of racism.
**S**trengthen families through wrap‐around services, cultural connection and activities	Strong resilient women and families Family preservation and restoration Reduction in youth crime and juvenile detention First Nations children commence school ready to learn and with the same educational opportunities as non‐Indigenous children.
**E**mbed First Nations community control and governance	Physical, social, cultural and economic wellbeing for First Nations communities

## 6 National First Nations Governance and Authorisation of the Final Roadmap

In October 2023, the draft Roadmap was distributed to First Nations organisations, ACCHOs, and peak bodies for feedback and review, before a First Nations only meeting. Facilitated by author RW (Kalkadunga, Djkunde), attendance included representatives from Rhodanthe Lipsett Indigenous Midwifery Charitable Fund, Secretariat of National Aboriginal and Islander Child Care (SNAICC), National Aboriginal Community Controlled Health Organisation (NACCHO), as well as individual First Nations midwifery leaders. This step marked the point at which the Roadmap transitioned from an evidence‐based proposal to a First Nations authorised and endorsed national framework, ready for enactment under First Nations governance, with governments required to align and respond.

## 7 Co‐Creation Phase 2: Indigenist, Iterative and Sovereignty‐Led Refinement (2025–2026)

**FIGURE 5:**
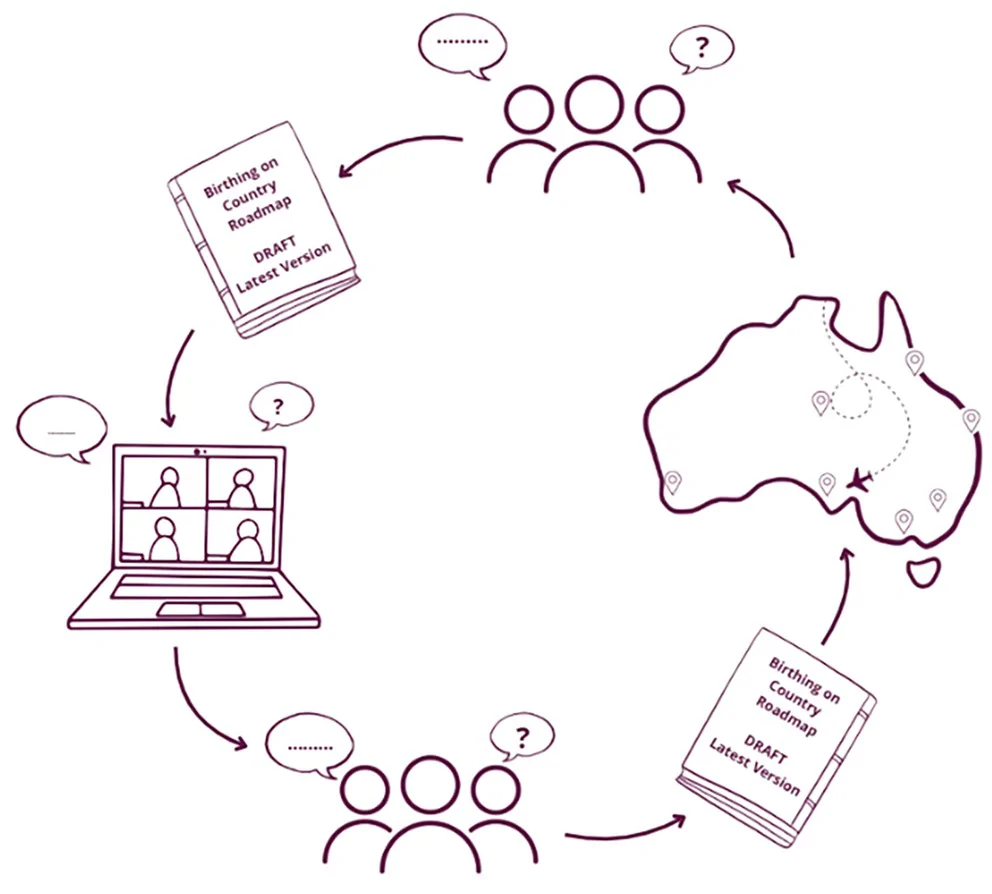
Co‐creation Phase 2: An iterative cyclical process.

In readiness to present the Roadmap to the Commonwealth Department of Health, Disability and Ageing, as well as state and jurisdictional health ministers, the Molly Institute garnered formal endorsement for the Roadmap from a range of ACCHOs, NACCHOs, the Congress of Aboriginal and Torres Strait Islander Nurses and Midwives (CATSINaM), medical peak bodies, health services, non‐government organisations, tertiary education centres and government departments. The Roadmap was disseminated at four large national conferences, to ensure a wide reach and engagement with the First Nations community, stakeholders and ACCHOs. Individuals who had considerable knowledge and expertise related to implementation of Birthing on Country services and knowledge of health systems were approached, including the relevant professional peak bodies (including peak bodies for First Nations health), government stakeholders, as well as First Nations and non‐First Nations leaders [39]. The Molly Institute facilitated over 40 meetings (in person or online) across all states and jurisdictions to garner feedback. Based on feedback received, the Roadmap was further refined [32] and five additional draft iterations were created. This crucial step enabled organisations and peak bodies to demonstrate their support and ensure the delivery of a collective vision for the Roadmap to government. The second phase of the co‐creation approach (Figure 5) involved an iterative, cyclical process of ongoing engagement with First Nations governance bodies, knowledge holders and community authorities, further feedback and refinement. Early feedback highlighted the need for the Roadmap to convey a sense of collective ownership and transparency regarding who supported the objectives and outcomes. At the time of publication of this article, the Roadmap has received formal endorsement by 21 organisations (11 of which are either First Nations peak bodies or community‐controlled) and a further three individuals nationally [30] (p. 2). Phase 2 ensured that the most recent evidence was included, and other important considerations that emerged from First Nations stakeholders were addressed (examples included maintaining the cultural integrity of Birthing on Country initiatives, ensuring the cultural legitimacy of the proposed National Taskforce [30] (p. 12), and inclusion of recommendations addressing land rights, for the purpose of health service infrastructure development). The outcome of this process led to a final *National Roadmap for Birthing on Country Services 2026–2036*, which is available online [30], and will be formally launched at a national gathering in September this year.

## 8 The Final Roadmap for Birthing on Country Services, 2026–2036

The final Roadmap [30] is a shared vision towards achieving self‐determination and equity in healthcare delivery for First Nations communities. It provides the whole of government with evidence‐based and practical strategies to expand and upscale Birthing on Country services. The Roadmap recommends a National Taskforce and a National Support Centre to solve complex legislative and systemic barriers to reform, such as ongoing insurance cover issues restricting ACCHOs from employing midwives who provide intrapartum care [28]. Growing the First Nations midwifery workforce is not merely an issue of workforce reform [40], but an act of cultural restoration and revival of interrupted birthing Law. The foundational co‐creation process of the Roadmap demonstrates that to optimise communication with clinicians and policy makers and change practice, researchers must do more than publish evidence in journals for policy creation and knowledge translation [41]. Moving forward, the Roadmap continues to be a living document that incorporates feedback and embeds ongoing co‐creation in its shared vision for equitable health outcomes and care.

A lesson of the Closing the Gap strategy has been that a lack of First Nations input led to slow progress and ineffective health policy. Strategic directions aiming to address health and social equity for First Nations communities need to be informed by sovereign First Nations voices [42]. As a First Nations maternal‐and‐infant health strategic direction, the Roadmap is novel in its co‐creation approach, which goes far beyond tokenism [31, 35]. Early engagement with key First Nations stakeholders (individuals, organisations, ACCHOs or peak bodies) was important to agree on processes, decision making and to develop a shared vision moving forward. A key message from First Nations knowledge holders was that the Roadmap needed to be ‘collectively owned’, with authority residing with First Nations organisations, ACCHOs, community women, Elders and matriarchs who have contributed to its development, whereby cultural legitimacy has been treated as equal to clinical legitimacy, and the authority of Elders, Leaders and matriarchs are addressed.

Birthing on Country was first recommended in policy 15 years ago; however, the majority of First Nations women remain without access, and implementation has not been systematic [6, 27] Three ACCHOs have so far overcome barriers to delivering community‐based Birthing on Country services wrap‐around support for women and their families across the entire childbearing continuum and beyond [43, 44]. Others are striving to gain resourcing and support to do the same. Barriers to research–practice translation and evidence‐based scale‐up including ongoing colonial governance structures that restrict First Nations self‐determination, funding constraints or inadequate financial incentives, lack of technical expertise and political resistance to change [41]. The Roadmap aims to go further in addressing the structural reform that has not been addressed in previous directives or policy locally and nationally. What this process uniquely engaged with is the need to transcend the current legislation, policies and practices that prevent First Nations ‘Nation Building’ [30] (p. 14), which requires an enhanced level of self‐governance and self‐determination [45]. First Nations scholars argue that this is what is required for optimal health and wellbeing outcomes [45]. If we are ever to achieve Closing the Gap's campaign objectives, full restoration of First Nation sovereign capacities can no longer be subverted, and we must create the political conditions for the Rights of Indigenous People to materialise in practice.

## 9 Conclusion

Forty years of strong community advocacy for the rights of First Nations women, and the return of birthing and maternal–infant health services to community control, have led to the co‐creation of the *National Roadmap for Birthing on Country Services 2026–2036*. This effort has continued despite decades of failure of policy, strategies and the maternity system to achieve equity for First Nations women and babies [10, 11]. The Roadmap builds on work arising from the Birthing on Country movement in Australia, drawing on a strong evidence base that demonstrates the benefits of Birthing on Country programs. Birthing on Country is not an innovation within the Australian health system; it is the restoration of sovereign First Nations birthing law and practice that predates and exceeds the authority of colonial institutions. Intergenerational cultural and ancestral knowledge have generously informed a step‐by‐step guide to achieve national restoration, resurgence and scaling up of Birthing on Country models of care. This Roadmap is a call to action to recognise Birthing on Country as a human right that must be governed by First Nations authority, with non‐Indigenous systems accountable to that leadership.

## Author Contributions

**Res McCalman and Anneka J. Bowman:** conducted data curation, formal analysis, methodology, investigation, project administration, resource provision, software, supervision at different (but equal) times through the project, validation and writing the original draft. **Roianne West:** conducted conceptualisation, methodology, led the national gatherings, and initial co‐creation, supervision and writing – review and editing. **Kristie Watego:** conducted conceptualisation and supervision, writing – review and editing. **Jyai Allen:** conducted data curation, formal analysis and project administration of Phase 1, as well as writing – review and editing. **Claire Clack:** conducted supervision, resources and writing – review and editing. **Sue Kildea and Sue Kruske:** conducted conceptualisation, funding acquisition, investigation and supervision. **Sue Kildea** additionally conducted writing – editing. **Melanie Briggs, Cleone Wellington and Rebecca Coddington:** all conducted conceptualisation, validation and writing – editing and review. **John D. Boffa and Marah Prior:** both conducted conceptualisation, administration during Phase 1, and writing – review and editing. **Yu Gao, Isabella Garti, Sascha Kowalenko, Sarah Ireland, Catherine Austin, Bettina Chaseling, Kelsie Kahl, Sarah Khaw and Emily Armstrong:** all conducted resourcing, data curation and writing – review and editing. **Donna Hartz and Yvette Roe:** both conducted conceptualisation and writing – editing and review. **Yvette Roe:** additionally provided cultural oversight, and management, data curation, funding acquisition, investigation, methodology, supervision, validation, visualisation and writing – review and editing.

## Funding

This work was supported by the National Health and Medical Research Council (2020/GNT1197110).

## Disclosure

Not commissioned; externally peer reviewed. Figure 2 was created with the use of AI technology and then modified by our team.

## Conflicts of Interest

The authors declare no conflicts of interest.

## Supporting information


**Data S1:** CONSIDER Statement.

## Data Availability

The authors have nothing to report.

## References

[1] P. Daylight and M. Johnstone , Women's Business: Report of the Aboriginal Women's Task Force (Commonwealth of Australia, 1986).

[2] B. Carter , E. Hussen , L. Abbott , et al., “Borning: Pmere Laltyeke Anwerne Ampe Mpwaretyeke; Congress Alukura by the Grandmother's Law: A Report Prepared by the Central Australian Aboriginal Congress,” Australian Aboriginal Studies 1, no. 2 (1987): 2–33.

[3] Y. Roe , M. Briggs , C. Buzzacott , D. Hartz , J. Sherwood , and S. Kildea , “Returning Birthing Services to Communities and Aboriginal Control: Aboriginal Women of Shoalhaven Illawarra Region Describe How Birthing on Country Is Linked to Healing,” Journal of Indigenous Wellbeing 5, no. 1 (2020): 58–71.

[4] S. Hickey , Y. Roe , S. Ireland , et al., “A Call for Action That Cannot Go to Voicemail: Research Activism to Urgently Improve Indigenous Perinatal Health and Wellbeing,” Women and Birth 34, no. 4 (2021): 303–305. 33935005 10.1016/j.wombi.2021.03.011

[5] S. Kildea , F. Magick Dennis , and H. Stapleton , Birthing on Country: Workshop Report (Maternity Services Inter Jurisdictional Committee for the Australian Health Ministers' Advisory Council, 2012).

[6] Central Australian Aboriginal Congress and Molly Wardaguga Research Centre , Best Start to Life: A National Gathering Report (Charles Darwin University, 2023).

[7] I. Ponomareva , L. Hatte , J. Kemp , M. Wallace , and C. McLennan , “The Archaeology of Sacred Womens' Business in Australia: A Holocene History From the Central Queensland Highlands,” Archaeology in Oceania 59, no. 2 (2024): 333–349.

[8] K. Adams , S. Faulkhead , R. Standfield , and P. Atkinson , “Challenging the Colonisation of Birth: Koori Women's Birthing Knowledge and Practice,” Women and Birth 31, no. 2 (2018): 81–88. 28803884 10.1016/j.wombi.2017.07.014

[9] D. Hartz and J. Sherwood , “Midwives Working With Aboriginal and Torres Strait Islander Women,” in Midwifery: Preparation for Practice, ed. S. Pairman , S. Tracy , H. Dahlen , and L. Dixon (Elsevier, 2019), 158–174.

[10] N. Sivertsen , T. Johnson , G. Mehus , T. S. M. Ness , S. Smith , and J. McGill , “What Is Known About Indigenous Women's Dissatisfaction of Birthing Experiences in Mainstream Maternity Hospitals in Australia, Aotearoa, Canada, US, Kalaallit Nunaat and Sápmi? A Systematic Scoping Review,” Frontiers in Public Health 13 (2025): 1495197. 40190765 10.3389/fpubh.2025.1495197 PMC11970129

[11] Australian Government, Australian Institute of Health and Welfare , Aboriginal and Torres Strait Islander Mothers and Babies [Website]. Cat. no: PER 120. (AIHW, 2023).

[12] H. Keedle , W. Keedle , and H. G. Dahlen , “Dehumanized, Violated, and Powerless: An Australian Survey of Women's Experiences of Obstetric Violence in the Past 5 Years,” Violence Against Women 30, no. 9 (2024): 2320–2344, 10.1177/10778012221140138. 36452982 PMC11145922

[13] C. Bond and D. Singh , “More Than a Refresh Required for Closing the Gap of Indigenous Health Inequality,” Medical Journal of Australia 212, no. 5 (2020): 198–199. 32030749 10.5694/mja2.50498

[14] R. Lovett , R. Jones , and B. Maher , “The Intersection of Indigenous Data Sovereignty and Closing the Gap Policy in Australia,” in Indigenous Data Sovereignty and Policy, ed. D. Rodriguez‐Lonebear , M. Walter , S. Russo Carroll , and T. Kukutai (Taylor & Francis, 2020), 36–50.

[15] Commonwealth of Australia , Commonwealth Closing the Gap 2025 Annual Report and 2026 Implementation Plan (Commonwealth of Australia, 2026).

[16] P. McCalman , D. Forster , M. Newton , F. McLardie‐Hore , and H. McLachlan , ““Safe, Connected, Supported in a Complex System.” Exploring the Views of Women Who Had a First Nations Baby at One of Three Maternity Services Offering Culturally Tailored Continuity of Midwife Care in Victoria, Australia. A Qualitative Analysis of Free‐Text Survey Responses,” Women and Birth 37, no. 6 (2024): e641–e651. 10.1016/j.wombi.2024.01.009 38302389

[17] Queensland Health , Growing Deadly Families: Aboriginal and Torres Strait Islander Maternity Services Strategy 2019–2025 (Queensland Health, 2019), https://www.health.qld.gov.au/__data/assets/pdf_file/0030/932880/Growing‐Deadly‐Families‐Strategy.pdf.

[18] Australian Government, Department of Health , National Women's Health Strategy, 2020‐2030 (Australian Government Department of Health and Aged Care, 2019), https://www.health.gov.au/sites/default/files/documents/2021/05/national‐women‐s‐health‐strategy‐2020‐2030_0.pdf.

[19] Department of Health , Koolin Balit: Victorian Government Strategic Directions for Aboriginal Health 2012–2022 (State of Victoria, Department of Health, 2012), https://www.health.vic.gov.au/sites/default/files/migrated/files/collections/policies‐and‐guidelines/1/1307014_koolin_balit_jul13_web‐‐‐pdf.pdf.

[20] Tasmanian Government, Department of Health , “Midwifery Matters: Tasmanian Midwifery Workforce Strategy 2025–2030 Consultation Draft,” (2025), https://www.health.tas.gov.au/sites/default/files/2025‐09/midwifery_matters_‐_tasmania_midwifery_workforce_strategy.pdf.

[21] COAG Health Council , Woman‐Centred Care: Strategic Directions for Australian Maternity Services (Department of Health, 2019), https://www.health.gov.au/sites/default/files/documents/2019/11/woman‐centred‐care‐strategic‐directions‐for‐australian‐maternity‐services.pdf.

[22] S. Kildea , Y. Gao , S. Hickey , et al., “Effect of a Birthing on Country Service Redesign on Maternal and Neonatal Health Outcomes for First Nations Australians: A Prospective, Non‐Randomised, Interventional Trial,” Lancet Global Health 9, no. 5 (2021): e651–e659. 33743199 10.1016/S2214-109X(21)00061-9

[23] B. O'Dea , Y. Roe , Y. Gao , et al., “Breaking the Cycle: Effect of a Multi‐Agency Maternity Service Redesign on Reducing the Over‐Representation of Aboriginal and Torres Strait Islander Newborns in Out‐Of‐Home Care: A Prospective, Non‐Randomised, Intervention Study in Urban Australia,” Child Abuse & Neglect 149 (2024): 106664. 38354600 10.1016/j.chiabu.2024.106664

[24] Y. Gao , Y. Roe , S. Hickey , et al., “Birthing on Country Service Compared to Standard Care for First Nations Australians: A Cost‐Effectiveness Analysis From a Health System Perspective,” Lancet Regional Health – Western Pacific 34 (2023): 100722. 37283966 10.1016/j.lanwpc.2023.100722 PMC10240371

[25] S. Kildea , S. Hickey , L. Barclay , et al., “Implementing Birthing on Country Services for Aboriginal and Torres Strait Islander Families: RISE Framework,” Women and Birth 32, no. 5 (2019): 466–475. 31279713 10.1016/j.wombi.2019.06.013

[26] M. Briggs , “Birthing On Country is a Sovereign Right For Indigenous Parents,” 2022, https://indigenousx.com.au/birthing‐on‐country‐is‐a‐sovereign‐right‐for‐indigenous‐parents/.

[27] H. McLachlan , M. Newton , F. McLardie‐Hore , P. McCalman , and D. Forster , “Improving Outcomes for First Nations Mothers and Babies in Australia Through Culturally Safe Continuity of Midwifery Care: The Time for Scale‐Up Is Now!,” EClinicalMedicine 61 (2023): 102093. 37483550 10.1016/j.eclinm.2023.102093 PMC10362253

[28] Y. Roe , J. Allen , P. Haora , et al., “Enabling the Context for Aboriginal and Torres Strait Islander Community Controlled Birthing on Country Services: Participatory Action Research,” Women and Birth 37, no. 2 (2024): 368–378. 38097448 10.1016/j.wombi.2023.11.007

[29] United Nations (General Assembly) , “United Nations Declaration on the Rights of Indigenous Peoples,” (A/RES/61/295), resolution/adopted by the General Assembly, 2007.

[30] Molly Wardaguga Institute for First Nations Birth Rights , National Roadmap for Birthing on Country Services 2026–2036. Version 6 (MRWI: Charles Darwin University, 2025).

[31] S. L. Sherriff , H. Miller , A. Tong , et al., “Building Trust and Sharing Power for Co‐Creation in Aboriginal Health Research: A Stakeholder Interview Study,” Evidence & Policy 15, no. 3 (2019): 371–392.

[32] D. M. Agnello , V. Anand‐Kumar , Q. An , et al., “Co‐Creation Methods for Public Health Research—Characteristics, Benefits, and Challenges: A Health CASCADE Scoping Review,” BMC Medical Research Methodology 25, no. 1 (2025): 60. 40050729 10.1186/s12874-025-02514-4 PMC11884017

[33] L.‐I. Rigney , “Internationalization of an Indigenous Anticolonial Cultural Critique of Research Methodologies: A Guide to Indigenist Research Methodology and Its Principles,” Wicazo Sa Review 14, no. 2 (1999): 109–121.

[34] M. Rishard , K. Rajaratne , K. Jayasingha , H. Senanayake , and M. S. D. Wijesinghe , “Cocreation and Codesign in Maternal Health: Processes, Outcomes and Implementation Challenges,” *BMJ Quality and Safety*, ahead of print, February 20, 2026, 10.1136/bmjqs-2025-019030. PMC13638332 41720630

[35] S. Bateman , M. Arnold‐Chamney , S. Jesudason , et al., “Real Ways of Working Together: Co‐Creating Meaningful Aboriginal Community Consultations to Advance Kidney Care,” Australian and New Zealand Journal of Public Health 46, no. 5 (2022): 614–621. 35797091 10.1111/1753-6405.13280

[36] T. Fitzsimmons , M. S. Yates , R. Jordan , and V. J. Callan , “Co‐Creating Impact: Positioning Indigenous Knowledge Holders as Expert Researchers,” Equality, Diversity and Inclusion: An International Journal 45, no. 3 (2024): 538–553.

[37] S. Kildea and V. Van Wagner , ‘Birthing on Country’ Maternity Service Delivery Models: A Rapid Review: An Evidence Check Rapid Review Brokered by the Sax Institute (Maternity Services InterJurisdictional Committee for the Australian Health Ministers' Advisory Council, 2013), https://www.saxinstitute.org.au/wp‐content/uploads/Birthing‐on‐Country1.pdf.

[38] NACCHO , “Aboriginal Community Controlled Health Organisations (ACCHOs),” [website], https://www.naccho.org.au/aboriginal‐community‐controlled‐health/.

[39] M. Vogelsang , L. McCaffrey , G. C. Ryde , M. Verloigne , and P. Dall , “Preparing for co‐Creation: A Roadmap for Planning a co‐Creation Initiative From a Case Study on Sedentary Behaviour in Scottish SMEs—A Health CASCADE Study,” Public Health 242 (2025): 157–164. 40080987 10.1016/j.puhe.2025.02.036

[40] C. S. E. Homer , K. Small , C. Watrton , et al., “Midwifery Futures—Building the Future Australian Midwifery Workforce,” (2024).

[41] K. Martin , Z. Mullan , and R. Horton , “Overcoming the Research to Policy Gap,” Lancet Global Health 7 (2019): S1–S2. 30857615 10.1016/S2214-109X(19)30082-8

[42] Lowitja Institute , “Close the Gap Campaign Report 2025: Agency, Leadership, Reform: Ensuring the Survival, Dignity and Wellbeing of First Nations Peoples,” The Close the Gap Campaign Alliance Group, (2025), https://lowitja.org.au/wp‐content/uploads/2025/03/Close‐the‐Gap‐Report‐2025_v5‐online_FINAL.pdf.

[43] Y. Gao , S. Kildea , R. Coddington , et al., “Effect of the Implementation of a Birthing on Country Service at a Rural Site, Waminda, Compared With Standard Care for First Nations Australians: A Prospective, Non‐Randomised, Interventional Trial,” Lancet Regional Health ‐ Western Pacific 67 (2026): 101796. 41657851 10.1016/j.lanwpc.2025.101796 PMC12873586

[44] P. Haora , Y. Roe , S. Hickey , et al., “Developing and Evaluating Birthing on Country Services for First Nations Australians: The Building on Our Strengths (BOOSt) Prospective Mixed Methods Birth Cohort Study Protocol,” BMC Pregnancy and Childbirth 23, no. 1 (2023): 77. 36709265 10.1186/s12884-022-05277-8 PMC9883816

[45] D. Rigney , S. Bignall , A. Vivian , and S. Hemming , Indigenous Nation Building and the Political Determinants of Health and Wellbeing: Discussion Paper (Lowitja Institute, 2022).

