# Supplementary material for: Forty Years in the Making: Reporting on the Co‐Creation of a National Roadmap for Birthing on Country Services for the Best Start to Life
Source: Med J Aust. 2026 Sep 1;224(9):e70282. doi: 10.5694/mja2.70282 (PMC13531727; doi:10.5694/mja2.70282)
Supplement: Supplementary file 1 [file MJA2-224-0-s001.pdf]

Supporting Information

Supplementary material

This appendix was part of the submitted manuscript and has been peer reviewed.
It is posted as supplied by the authors.

Appendix to: McCalman R, Bowman AJ, West R, et al. Forty years in the making: Reporting on the Co-creation of a National Roadmap for Birthing on Country services for the Best Start to Life. *Med J Aust* 2026; doi: 10.5694/mja2.70282.

CONSIDER Statement Template

Guest Editors of the 2026 *Indigenous Health Special Issue* acknowledge the Indigenous expertise that informed the establishment of the CONSolIDated critERia for strengthening the reporting of health research involving Indigenous Peoples (CONSIDER) statement [1].

Authors should indicate how they have supported ethical publishing and reporting practices by providing the details of the research practices aligned with this publication in accordance with the CONSIDER statement. The reporting should not exceed two pages. This reporting will be published as online supplementary information. Detailed items can be accessed in the publication:

https://bmcmedresmethodol.biomedcentral.com/articles/10.1186/s12874-019-0815-8

**Governance**
We report in this manuscript that the roadmap has received endorsement from 11 Aboriginal community controlled, or First Nations-led organisations (ACCHOs, First Nations peak bodies, or First Nations led research institutes) and a personal endorsement from the Australia's sole First Nations Director of Midwifery. A key message from First Nations stakeholders was that the Roadmap needed to be 'collectively owned', with authority residing with First Nations organisations, community women, Elders and matriarchs who have contributed to its development, whereby cultural legitimacy has been treated as equal to clinical legitimacy, and the authority of Elders, Leaders and matriarchs are addressed.<br>Co-creation, as applied in this Roadmap, is an Indigenous self-determination methodology as it centres Indigenous authority in knowledge, decision-making and governance by making space for First Nations women's leadership. All knowledge generated through this process is governed under Indigenous Data Sovereignty principles, ensuring First Nations ownership, control and application.
**Prioritization**
As a manifestation of the Birthing on Country social justice movement, which builds on four decades of community advocacy, and evidence of the benefit of Birthing on Country services for First Nations women, and the need for sovereignty and self-determination in the creation of First Nations health policy, this manuscript addresses an important Aboriginal and Torres Strait Islander community priority. First Nations stakeholders have expressed the need for this Roadmap as a response to the current physical, social, economic and cultural environments that perpetuate the perinatal health inequities experienced by First Nations communities today, which stem from colonisation, racism and social injustice. Additionally, strategic health policy that is genuinely informed by sovereign First Nations voices is needed for improved health and wellbeing of First Nations people, hence why it is important to report on the development of such a policy.<br>This manuscript reports on the co-creation of a First Nations maternal–infant health strategy that was a key deliverable of a large national gathering. Central Australian Aboriginal Congress (CAAC) was the co-host with the Molly Wardaguga Research Institute. The working relationship between the Molly Wardaguga Institute and CAAC is formalised through a multi-institutional agreement, as well as longstanding collaborative working relationships. Specific to this work, there was a joint working group in the planning and delivery of the conference that would meet fortnightly i.e. developing the program, guest speakers, cultural activities, media coordination, logistics support.<br>Other organisations have all been instrumental in the co-creation of the Roadmap, and have been acknowledged accordingly without formal partnership or agreement.
**Relationships (Indigenous stakeholders/participants and Research Team**
The Molly Wardaguga Institute for First Nations Birth Rights is a leading institute in First Nations maternal and infant health. First author R. McCalman is a Noongar midwife with research expertise on First Nations women's maternity models of care, co-first author A. Bowman has worked for over a decade supporting research to improve care for First Nations families through the perinatal period. Senior author Y. Roe has more than 30 years' experience working in Indigenous health and is the Director of the Molly Wardaguga Research Institute. The research team is comprised of perinatal health researchers with experience working alongside communities to improve maternity care for First Nations women, industry experts, policy makers and community-based women who are partners of the Molly Wardaguga Institute, and who are currently implementing Birthing on Country services in their communities.

Methodologies
As discussed in the manuscript, co-creation processes are innovative, creative and inclusive and aim for communal and societal benefits to be shared. Genuine co-creation is principled upon collaboration, participation, empowerment and agency, aiming to produce innovative, valid and impactful solutions for specific contexts. Building on participatory governance, co-creation addresses imbalances of power in decision making processes and is an ethical mechanism for making space for First Nations women's leadership, a conscious 'pacing' of processes that enable relationship building and maintenance. The Roadmap was also underpinned by the RISE Implementation Framework, which is grounded in First Nations ways of knowing, being and doing, with Western evidence positioned as supportive, rather than authoritative. The manuscript outlines the process of stakeholder engagement.
**Participation**
We report on the development of the Roadmap, which included representatives from Rhodanthe Lipsett Indigenous Midwifery Charitable Fund, Secretariat of National Aboriginal and Islander Child Care (SNAICC), the National Aboriginal Community Controlled Health Organisation (NACCHO), as well as individual First Nations midwifery leaders, representatives from ACCHOs, community representatives, family support and Aboriginal health workers.
**Capacity**
Steering the narrative at the Best Start to Life gathering were the voices of First Nations women and organisations from urban, rural, remote and very remote Australia, who have, or are planning to, deliver Birthing on Country services, including the community-based members of the authorship team on this manuscript. The gathering showcased innovative, translational research, and leading practice changes from around the country, attracting significant national interest. This manuscript is led by a First Nations early career researcher, and this manuscript details the ongoing process of relationship maintenance with First Nations stakeholders in the development of this Roadmap.
**Analysis and interpretation**
This manuscript calls for action to recognise Birthing on Country as an Indigenous right that must be governed by First Nations authority, with non-Indigenous systems accountable to that leadership.
**Dissemination**
The manuscript details that the Roadmap was disseminated at four large national conferences, to ensure wide reach and engagement with the First Nations community, stakeholders and ACCHOs. The Roadmap will be formally launched in September 2026 at a First Nations-led maternal and child health conference.<br>As a mechanism for translating evidence into policy and action, 'Co-creation' fosters enhanced engagement from a broad range of stakeholders and collaborators to produce inclusive and democratic solutions to complex problems, which in this instance, are the perpetual perinatal inequities produced by the current maternity system, and slow national implementation of Birthing on Country services.

Reference

1 Huria T, Palmer SC, Pitama S, et al. Consolidated criteria for strengthening reporting of health research involving indigenous peoples: the CONSIDER statement. *BMC Med Res Methodol* 2019; 19: 17.
